# Blood flow restriction *Ex*ercise in the perioperative setting to *Prev*ent loss of muscle mass in patients with pancreatic, biliary tract, and liver cancer: study protocol for the PREV-Ex randomized controlled trial

**DOI:** 10.1186/s13063-024-08207-5

**Published:** 2024-06-04

**Authors:** Poorna Anandavadivelan, Daniele Cardinale, Rune Blomhoff, Berit Sunde, Kristoffer Lassen, Dyre Kleive, Christian Sturesson, Stefan Gilg, Truls Raastad, Sara Mijwel

**Affiliations:** 1 Department of Neurobiology, Care Sciences and Society, Division of Nursing, Karolinska Institute, Stockholm, Sweden; 2 The Åstrand Laboratory, The Swedish School of Sport and Health Sciences, Stockholm, Sweden; 3 Department of Nutrition, Institute of Basic Medical Sciences, University of Oslo, Oslo, Norway; 4 Division of Surgery, Department of Clinical Science, Intervention and Technology, Karolinska Institute, Solna, Sweden; 5 Department of Upper Abdominal Diseases, Karolinska University Hospital, Stockholm, Sweden; 6 Department of Hepatobiliary and Pancreatic Surgery, Oslo University Hospital, Oslo, Norway; 7 Department of HPB Surgery, Karolinska University Hospital, Stockholm, Sweden; 8 Department of Physical Performance, Norwegian School of Sport Sciences, Oslo, Norway

**Keywords:** Blood flow restriction training, Pancreatic cancer, Biliary tract cancer, Liver cancer, Prehabilitation, Rehabilitation, Skeletal muscle mass, Muscle atrophy

## Abstract

**Background:**

Patients diagnosed with pancreatic, biliary tract, and liver cancer often suffer from a progressive loss of muscle mass. Given the considerable functional impairments in these patients, high musculoskeletal weight loads may not be well tolerated by all individuals. The use of blood-flow restricted resistance training (BFR-T) which only requires low training loads may allow for a faster recovery of muscle due to avoidance of high levels of mechanical muscle stress associated with high-load resistance exercise. This study aims to investigate whether BFR-T can prevent or slow down the loss of skeletal muscle mass and enhance the functional capacity and mental health of patients with pancreatic, biliary tract, and liver cancer.

**Methods:**

The PREV-Ex exercise trial is a multicenter two-armed randomized controlled trial. Patients will be randomized to an exercise program consisting of home-based low-load BFR-T during a combined pre- and postoperative period for a total of 6–10 weeks (prehabilitation and rehabilitation), or to a control group. Protein supplementation will be given to both groups to ensure adequate protein intake. The primary outcomes, skeletal muscle thickness and muscle cross-sectional area, will be assessed by ultrasound. Secondary outcomes include the following: (i) muscle catabolism-related and inflammatory bio-markers (molecular characteristics will be assessed from a vastus lateralis biopsy and blood samples will be obtained from a sub-sample of patients); (ii) patient-reported outcome measures (self-reported fatigue, health-related quality of life, and nutritional status will be assessed through validated questionnaires); (iii) physical fitness/performance/activity (validated tests will be used to evaluate physical function, cardiorespiratory fitness and maximal isometric muscle strength. Physical activity and sedentary behavior (assessed using an activity monitor); (iv) clinical outcomes: hospitalization rates and blood status will be recorded from the patients’ medical records; (v) explorative outcomes of patients’ experience of the exercise program which will be evaluated using focus group/individual interviews.

**Discussion:**

It is worthwhile to investigate new strategies that have the potential to counteract the deterioration of skeletal muscle mass, muscle function, strength, and physical function, all of which have debilitating consequences for patients with pancreatic, biliary tract, and liver cancer. The expected findings could improve prognosis, help patients stay independent for longer, and possibly reduce treatment-related costs.

**Trial registration:**

ClinicalTrials.gov NCT05044065. Registered on September 14, 2021.

## Administrative information

Note: the numbers in curly brackets in this protocol refer to SPIRIT checklist item numbers. The order of the items has been modified to group similar items (see http://www.equator-network.org/reporting-guidelines/spirit-2013-statement-defining-standard-protocol-items-for-clinical-trials/).

**Table:** 

Title {1}	Blood flow restriction *Ex*ercise in the perioperative setting to *Prev*ent loss of muscle mass in patients with pancreatic, biliary tract, and liver cancer: study protocol for the PREV-Ex randomized controlled trial
Trial registration {2a and 2b}.	NCT05044065, ClinicalTrials.gov: Blood Flow Restriction Exercise in the Perioperative Setting to Prevent the Loss of Muscle Mass in Patients with Pancreatic, Biliary Tract, and Liver Cancer—the PREV-Ex Randomized Controlled Trial.
Protocol version {3}	11/03/2024 Version 1.1.
Funding {4}	Financial support: The Swedish Research Council Karolinska Institute Research Foundations The Cancer Research Funds of Radiumhemmet
Author details {5a}	1 Department of Neurobiology, Care Sciences and Society, Division of Nursing, Karolinska Institute, Stockholm, Sweden 2 The Åstrand Laboratory, The Swedish School of Sport and Health Sciences, Stockholm, Sweden 3 Department of Nutrition, Institute of Basic Medical Sciences, University of Oslo, Oslo, Norway 4 Division of Surgery, Department of Clinical Science, Intervention and Technology, Karolinska Institute, Solna, Sweden 5 Department of Upper Abdominal Diseases, Karolinska University Hospital, Sweden 6 Department of Hepatobiliary and Pancreatic Surgery, Oslo University Hospital, Oslo, Norway 7 Department of HPB Surgery, Karolinska University Hospital, Stockholm, Sweden 8 Department of Physical Performance, Norwegian School of Sport Sciences, Oslo, Norway
Name and contact information for the trial sponsor {5b}	Karolinska University Hospital, Karolinska Universitetssjukhuset, Solna, 171 76 Stockholm The Norwegian School of Sport Sciences, Sognsveien 220, 0863 Oslo
Role of sponsor {5c}	The funders and study sponsors have no role in the study design, data collection or analysis, decision to publish, or preparation of the manuscript.

## Introduction

### Background and rationale {6a}

Patients with resectable pancreatic, biliary tract, or liver cancer are at increased risk of skeletal muscle loss attributed to several factors including surgery, disease burden, inactivity, and treatment [1, 2]. Skeletal muscle wasting occurs as early as 1 to 2 years before pancreatic cancer diagnosis and can lead to cachexia, a debilitating and ubiquitous condition characterized by an ongoing accelerated loss of skeletal muscle mass leading to progressive functional impairments [1, 3]. No drug or nutritional interventions have to date been proven effective against cachexia [4]. Given the complexity of cancer cachexia, its successful management will require an intervention with pleiotropic effects. Resistance exercise produces multifactorial and anabolic stimuli [5] and acts on several signaling pathways to antagonize muscle atrophy in cachexia. In cachectic mice, resistance training by ladder climbing counteracted muscle wasting and improved survival [6]. Moreover, some of the few randomized exercise trials in patients with pancreatic cancer showed that supervised resistance training was safe, feasible, and improved muscle strength [7] and quality of life [8]. Despite advanced disease, patients with pancreatic cancer are willing and interested in participating in an exercise intervention [9]. However, given the considerable functional impairments and muscle loss faced, high-load exercise may not be well tolerated by all individuals. One resistance training modality, namely blood flow restriction training (BFR-T), that incorporates low‐load resistance training with concurrent partial restriction of blood flow to the working muscles, induces substantial gains in muscle strength and muscle mass in both frail elderly [10] and in patients with myopathy [11]. Despite the beneficial effects on skeletal muscle, only one study explored BFR-T in patients with cancer demonstrating its feasibility and effectiveness in enhancing physical function with 4 weeks of pre-surgery intervention in patients with abdominal cancer [12]. Due to the low muscle loading, BFR-T could serve as a feasible exercise modality in patients with cancer that can be implemented safely both at home and in the clinic. Given the heightened risk of infection and reduced ability to travel during active treatment, a safe and effective home-based exercise intervention is preferred by patients. Furthermore, perioperative interventions like BFR-T could potentially minimize adverse events (AEs) and improve surgery-related outcomes. Minimal equipment, low-weight loads, or body weight loads are required to carry out BFR-T, making the future implementation of such a training strategy highly attractive.

### Objectives {7}

The *overall objective* of this randomized controlled trial is to examine if low load BFR-T can prevent muscle atrophy or mitigate its progression by studying changes in muscle mass and muscle signaling in patients with resectable pancreatic, biliary tract, or liver cancer.

Using a two-armed randomized controlled trial *the primary aim* of this project is to assess if a BFR-T program can preserve or increase skeletal muscle mass measured as thickness and cross-sectional area in patients with resectable pancreatic, biliary tract, or liver cancer.

The *secondary aims* are to investigate the effects of BRF-T on:

- Myogenic stem cells, mitochondrial function, capillarization, macrophage infiltration, and muscle catabolism, measured in skeletal muscle biopsies from a subgroup of voluntary participants
- Muscle strength, physical function, body composition, and physical activity levels
- Self-reported fatigue, health-related quality of life (HRQoL), and nutritional status
- The patients’ experience of the exercise program
- Hospitalization, prognostic markers, and different blood status concentrations

The use of BFR-T using low training loads may allow for a quick postoperative recovery by avoiding the high levels of mechanical muscle stress typically associated with high-load resistance exercise. Thus, BFR-T could serve as a feasible exercise modality in this patient population to mitigate the burden of cachexia. Moreover, the metabolic stress induced by BFR-T may have the potential to normalize the disrupted intramuscular milieu in cachectic muscle leading to a proper muscle regenerative response. Therefore, it is expected that the challenge imposed by BFR-T will:

- Stimulate muscle hypertrophy and thereby preserve or increase muscle mass
- Induce beneficial effects on skeletal muscle characteristics in terms of myogenic stem cell count and differentiation, basal autophagy activity, mitophagy/biogenesis, capillaries per muscle fiber, macrophage infiltration, and reduce the levels of catabolic markers
- Improve muscle strength, physical function, and physical activity levels
- Counteract an increase in self-reported fatigue and a decline in HRQoL
- Due to its proposed positive effects on skeletal muscle mass and quality, lead to lower hospitalization rates

### Trial design {8}

**Fig. 1:**
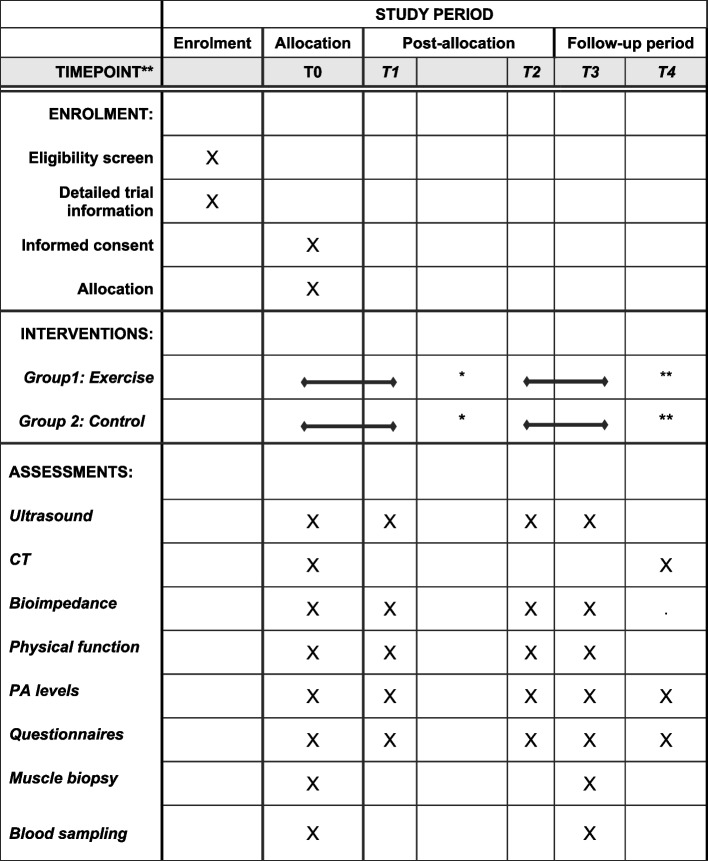
Schedule of enrolment, interventions, and assessments in the PREV-Ex trial*. T0 diagnosis or post neoadjuvant chemotherapy; T0–T1 Prehabilitation period 1–4 weeks; *Surgery to discharge from hospital (2–3 weeks); T2–T3 post-surgery training period 5–6 weeks; T3–T4 follow-up 6 months following surgery

This Prev-Ex is a multi-center, two-armed randomized controlled trial (RCT) with 1:1 allocation ratio, stratified for research site, and testing the superiority of the intervention. Patients diagnosed with resectable pancreatic, biliary tract, and liver cancer are randomized to an exercise program consisting of home-based low-load BFR-T during both a pre- and postoperative period consisting of a total of 6–10 weeks, or a control group. Besides the nutritional support offered as standard care for these patients, protein supplementation will be given to both groups to ensure adequate protein intake. See Fig. 1 for the modified SPIRIT study flow chart.

## Methods: participants, interventions, and outcomes

### Study setting {9}

Patients will be recruited from two sites: Karolinska University Hospital in Stockholm, Sweden, and Oslo University Hospital in Oslo, Norway. Testing of the participants’ physical fitness and body composition will take place in Stockholm at the research gym laboratory (Rörelseglädjelabbet), Karolinska University Hospital, and in Oslo at the exercise testing facilities at the Norwegian School of Sport Sciences.

### Eligibility criteria {10}

Inclusion criteria are as follows: 18 years or older, males or females, diagnosis of resectable pancreatic cancer, biliary tract cancer, or malignancies in the liver amendable for surgical resection, can maintain oral food intake during the intervention period, ECOG status < 2. Exclusion criteria include ongoing infection, serious neurological or cardiac disease, uncontrolled pain, pregnancy, difficulties breathing, and other contraindications to exercise.

### Who will take informed consent? {26a}

At both sites, a member of the medical staff will screen a potential patient before their hospital visit following the multidisciplinary meeting if eligible. At the hospital visit, the patient is provided with study information in written form, and is asked if a project manager from the research labs may contact the patient by phone. At the Swedish site, patients will, after a reasonable time for reflection, be contacted by a telephone call and will be asked if they are willing to participate. At the Norwegian site, patients will initially be informed about the study through a text message by the research team asking patients to answer whether they are willing to receive more information about the study or not. If the participant is interested to receive more information about the study,  a phone call is made to provide further information and answer questions. Subsequently another text message is sent where participants are asked to answer if they are willing to participate or not. The reason for the varying patient approach is that the Norwegian Regional committees for medical and health research ethics does not authorize recruitment by telephone calls, as this may be perceived as undue pressure to participate. Eligible patients who are willing to participate are invited to the study center to sign written informed consent and to undergo baseline measurements. Patients who choose not to participate in the PREV-Ex study are asked, but not required, to provide a reason for non-participation. The study will be conducted in full conformance with the principles of the “Declaration of Helsinki.”

### Additional consent provisions for collection and use of participant data and biological specimens {26b}

At the Norwegian site, participants are asked if they are willing to donate a muscle biopsy and blood samples, and for those patients who accept, an additional signature is obtained on the informed consent document. The material will be stored in a project-specific research biobank at the Norwegian School of Sport Sciences.

#### Interventions

### Explanation for the choice of comparators {6b}

Usual care group: The UC group will receive protein supplementation to ensure adequate protein intake and they will receive an activity tracker. Both the intervention and control groups receive protein supplements to ensure adequate intake and activity trackers to encourage regular physical activity. This setup aims to isolate the added benefits of BFR-T. While activity trackers might boost physical activity in the control group, they also reduce drop-out rates and contamination.

### Intervention description {11a}

#### Exercise intervention

The exercise intervention will consist of home-based low load blood flow restricted resistance training 4-5 times weekly during the 1–4-week prehabilitation period and 2–3 times per week during the 5–6-week rehabilitation period. During the initial baseline visit (T0), patients who are allocated to the exercise group will receive instructions about how to perform the BFR-T exercises. Blood flow restriction is induced by pressure cuffs connected to a tourniquet system (Occlude, Aarhus, Denmark). The cuff is wrapped around the proximal part of the legs and arms to partially restrict blood flow into the muscle and occlude blood flow out of the muscle and the cuff pressure will be set at 50% of arterial occlusion pressure (AOP). The individualized cuff pressure should not cause pain. During the preoperative phase, patients will perform three sets of 20–15–15 repetitions for each exercise during the first week and for the remaining pre-operative weeks the volume will be increased to three sets of 30–15–15 repetitions. During the rehabilitation phase post-surgery, three sets of 20/30–15–15 repetitions will be performed. During BFR-T, the cuff will be kept inflated and released immediately upon completion of the third set. The weight loads used during exercise will be very low using the individual’s own body weight and resistance bands will be used for two of the exercises. Exercises consist of a warmup (5 min walking with cuffs placed on the legs), lunges, squats, push-ups, seated row with a resistance band, and biceps curls with a resistance band. The exercises and the repetitions/sets will be adjusted according to the patient's functional capacity. The home-based sessions will be monitored/supervised digitally or through telephone calls once per week. The home-based exercise setup enables participants to avoid time-consuming travel and allows participants to exercise independently, without access to a gym facility. This may be especially important to consider during and after times of a pandemic considering that this patient population may be more sensitive to infections.

### Criteria for discontinuing or modifying allocated interventions {11b}

In case of adverse events (AEs) and serious adverse events (SAEs), which are severe or unexpected AEs that pose a threat to the participant's safety, discontinuation or modification of the intervention may occur. The severity, frequency, and relationship of AEs to the study intervention are considered. If the participant's condition worsens, modifications or discontinuation of the intervention may be appropriate. Moreover, if a participant voluntarily withdraws from the study or requests discontinuation of the intervention, their decision will be respected. New scientific evidence, either from the trial or external sources, indicating safety concerns or lack of efficacy may also lead to intervention modifications or discontinuation.

### Strategies to improve adherence to interventions {11c}

Patients are informed about the importance of adherence, potential benefits, and possible risks during the informed consent process. An effort has also been made to reduce the number and complexity of procedures to reduce the burden on participants, making it easier for patients to comply with the intervention. Timely feedback on participants’ progress and adherence is provided and monitoring/supervising the home-based exercise sessions through telephone calls/digitally will help track adherence to the exercise intervention and identify and address issues early, preventing them from becoming major barriers to adhere to the intervention. Patients will receive an activity tracker, Fitbit Inspire 2 (Fitbit Inc, subsidiary of Google LLC, San Francisco, CA, USA) on the wrist during all waking hours to monitor the activity levels. Protein supplement intake will be recorded through a protein intake log to track adherence.

### Relevant concomitant care permitted or prohibited during the trial {11d}

No concomitant care is prohibited during the trial.

### Provisions for post-trial care {30}

Each participant will receive an activity tracker (Fitbit Inspire 2, Fitbit Inc, subsidiary of Google LLC), and they will be allowed to retain it following the conclusion of the trial as an incentive for promoting ongoing physical activity. No harm is expected from the exercise trial since exercise has been shown to be safe and feasible in patients with cancer.

### Outcomes {12}

#### Primary outcome

The primary outcomes are skeletal muscle thickness and skeletal muscle cross-sectional area and will be determined by ultrasound at baseline (T0) and immediately (1–4 weeks) after the prehabilitation period (T1), after surgery prior to the rehabilitation period (T2) and after the 5–6-week postoperative rehabilitation period (T3). 

### Secondary outcomes

- ➢ Skeletal muscle molecular characteristics will be assessed in skeletal muscle vastus lateralis biopsy tissues from participants included in the study who agree to undergo the biopsy procedure (only at the Norwegian site: T0, T3).
-
  - ◦ Myogenic stem cell count
  - ◦ Autophagy
  - ◦ Capillary number per muscle fiber
  - ◦ Macrophage infiltration
  - ◦ Markers of skeletal muscle catabolism
- ➢ Patient-reported outcome measures (T0, T1, T2, T3, T4)
- ◦ Self-reported fatigue assessed by means of the validated European Organization for Research and Treatment of Cancer Fatigue questionnaire (EORTC QLQ-FA12).
- ◦ Health-related quality of life (HRQoL) assessed by means of the validated Quality of Life Core Questionnaire (EORTC-QLQ-C30)
- ◦ Nutritional status will be assessed by means of the validated Patient-Generated Subjective Global Assessment scale (PG-SGA).
- ➢ Physical fitness/performance/activity
-
  - ◦ Physical function determined by the short physical performance battery and a submaximal and maximal (ramp) cycle test to evaluate cardiorespiratory fitness and maximal isometric muscle strength evaluated in a standardized manner (T0, T1, T2, T3)
  - ◦ Objectively measured physical activity and sedentary behavior using the Fitbit activity monitor (T0, T1, T2, T3, T4)
- ➢ Clinical outcomes will be recorded retrospectively from the patient’s medical records (T4)
-
  - ◦ Hospitalization rates
  - ◦ Blood status
- ➢ Vital signs and anthropometrics
-
  - ◦ Body composition using bioimpedance analysis (T0, T1, T2, T3)
  - ◦ Body composition using dual x-ray absorptiometry (only at the Norwegian site: T0, T3)
  - ◦ Body composition will also be measured using computed tomography (CT) scans when performed as a routine measurement for the patients (T0, T4). Thus, no extra CT scans will be conducted for the purpose of this study to minimize exposure to radiation.
- ➢ Explorative outcomes

Patient’s experience of the exercise program, protein supplement, and activity tracker through focus group or individual interviews will take place sometime following the exercise intervention for a voluntary subset of the included participants in the exercise group.

### Participant timeline {13}

**Fig. 2:**
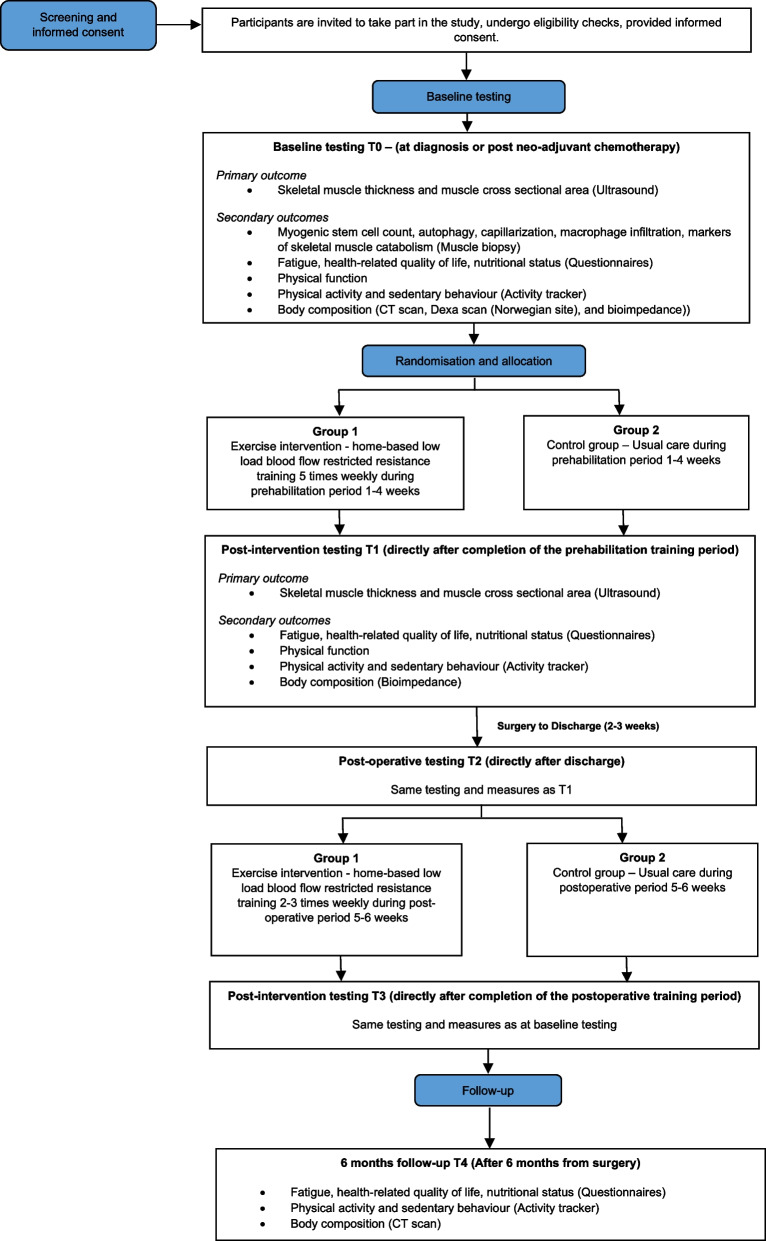
The modified SPIRIT study flow chart

Study outcomes will be measured at baseline (T0) and immediately (1–4 weeks) after the prehabilitation period (T1), after surgery prior to the rehabilitation period (T2), and after the 5–6-week postoperative rehabilitation period (T3). A follow-up assessment (T4) will take place 6 months following surgery. Patients with short time-to-surgery will only perform (T0) prior to surgery or will be included at (T2) for the rehabilitation period. Focus group or individual interviews will take place sometime following the exercise intervention. Hospitalization rates, prognostic- and blood markers will be assessed retrospectively at (T4) through the patients’ medical records. Baseline testing will begin immediately as participants are recruited and randomization will occur directly after the initial testing session (T0). Exercise training for the intervention group will start once participants have completed baseline testing. See Fig. 2 for an overview of the study design and measurement outcomes and time points.

### Sample size {14}

To achieve 80% power at an alpha level of 0.05 (two-tailed) from pre- to post-intervention based on a magnitude of change of 1 mm in skeletal muscle thickness [13], and a standard error of measurement of 0.8 mm [14], a total of 42 participants are required. According to previous findings [5], we anticipate that 25% of the participants may drop out of the study; therefore, a total of 52 participants will be required to confirm or refute our hypothesis.

### Recruitment {15}

Fifty-two patients with pancreatic, biliary tract, and liver cancer (26 in each group) will be recruited from two sites: Karolinska University Hospital in Stockholm, and Oslo University Hospital in Oslo, Norway, based on the inclusion and exclusion criteria. Due to the pandemic, the start of the inclusion of the first participant was delayed. Recruitment of the first participant was in May 2022 at the Swedish site and in February 2023 at the Norwegian site and is ongoing. The expected time of the final recruitment of participants from both sites is the end of 2024, and with that, the first phase (T0 to T3) of the project will be completed.

After a patient expresses interest, a member of the medical staff will provide detailed trial information and screen for eligibility as per the inclusion and exclusion criteria. Once the participants wish to volunteer for the study and after a reasonable time for reflection, the participants then sign the written informed consent form prior to randomization and intervention.

## Assignment of interventions: allocation

### Sequence generation {16a}

After baseline assessments, the participants are randomly allocated by the ALEA randomization software (ALEA Clinical Services B.V, 2023, The Netherlands) to either BFR-T or UC at a 1:1 ratio using a blocked computer-generated sequence.

### Concealment mechanism {16b}

Allocation concealment will be ensured as the ALEA randomization software will not release the randomization code until the patient has been recruited into the trial.

### Implementation {16c}

Immediately following baseline testing the research study team will enter information in the ALEA randomization software and inform the participant of the assigned intervention group.

## Assignment of interventions: blinding

### Who will be blinded {17a}

As this is an exercise intervention, participants, local study nurses, or investigators cannot be blinded to group assignment. Outcome assessors are not blinded to group allocation, except for assessors of muscle biopsy and blood samples who are blinded to group allocation. The health care providers and surgeons are not blinded to group allocation since they may discuss the intervention with the patients.

### Procedure for unblinding if needed {17b}

As this is an exercise intervention, participants cannot be blinded to group assignment and a participant’s allocated intervention will be revealed to the participant immediately following baseline testing.

## Data collection and management

### Plans for assessment and collection of outcomes {18a}

#### Study instruments

Skeletal muscle thickness and skeletal muscle cross-sectional area will be determined by ultrasound. Ultrasound serves as an imaging method capable of assessing the thickness and cross-sectional areas of surface-level muscles, such as the rectus femoris muscle. Its portability is a notable advantage. Numerous studies have validated the reliability of ultrasound in measuring the quadriceps muscle size in healthy individuals and patients in other settings [15].

- Self-reported fatigue will be assessed by means of the validated EORTC Fatigue questionnaire EORTC QLQ-FA12 [16, 17]. The EORTC QLQ-FA12 is a multidimensional instrument that measures various aspects of cancer-related fatigue, including overall, physical, emotional, and cognitive aspects. Fatigue scores obtained from the questionnaire are transformed into scales ranging from 0 to 100. Higher scores on the scales indicate a higher level of cancer-related fatigue. Missing data will be handled according to the guidelines provided in the EORTC scoring manual.
- HRQoL will be assessed by means of the validated EORTC-QLQ-C30 [15]. The EORTC QLQ-C30 is a 30-item questionnaire that assesses the quality of life in cancer patients with five multi-item functional scales (physical, role, cognitive, emotional, and social); one global quality of life scale; three symptom scales (fatigue, pain, and nausea/vomiting) and six single-items measuring symptoms common among patients with cancer in general (dyspnea, insomnia, appetite, constipation, and diarrhea) and financial impact. The HRQoL scores will be transformed into a scale ranging from 0 to 100 where higher scores for function and/or global QOL correspond to better HRQoL, while higher scores for symptoms represent worse problems. Missing data will be handled according to the guidelines provided in the EORTC scoring manual.
- Nutritional status will be assessed by means of the PG-SGA scale [18]. The PG-SGA has four components, nutritional status, the physical examination, disease/condition and metabolic demand assessment components, and medical history component (comprising weight history, food intake, nutrition impact symptoms, as well as activities and function component). All four components are self-reported by patients. A total score is calculated by summing the scores from the four components, where higher scores indicate worse nutritional status.
- Physical function will be assessed by the short physical performance battery that includes the side-by-side stand (10 s), the semi-tandem stand (10 s), tandem stand (s), gait speed test (s), and chair stand test — 5 times (s). A total score will be calculated for the battery of tests included in short physical performance. Muscle strength is being assessed by a hydraulic hand dynamometer (JAMAR, SAEHAN corporation, Chang-won, S. Korea), and isometric muscle strength tests measuring upper and lower limb muscle strength by leg extension and chest press using a power meter (Swedish site: HUR, Ab HUR Oy, Kokkolac, Finland. Norwegian site: Gym2000, Vikersund, Norway; Strain gauge: U2A 200, Hottinger Baldwin Mestechnik, Darmstadt, Germanuý (custom made amplifier); data acquisition: Labview, National Instruments, TX, USA (custom built)). Cardiovascular fitness is assessed, both predicted and maximal oxygen uptake (VO_2peak_), through the validated sub-maximal Ekblom-Bak test [19] and a ramp test on a cycle ergometer to exhaustion which has been shown to accurately predict VO_2peak_ [20], (LODE, Lode BV, Groningen, The Netherlands).

### Plans to promote participant retention and complete follow-up {18b}

The research team has successfully completed a large randomized controlled trial and has experience regarding patient recruitment and possible dropout rates and complications that can occur when conducting clinical exercise studies. Taking these types of unexpected factors into consideration, the inclusion rate is expected to be 3 patients per month meaning that the data collection will be completed at the end of 2024.

### Data management {19}

Data is collected and entered exclusively by the study research staff. All hard copies of forms are kept in a locked filing cabinet at Karolinska Institutet. Data are entered into computer files in a re-identifiable format (participant numbers only) and password protected. Participants will not be identified in the resulting manuscripts and reports. The data monitoring committee for the current study is made up of the research project staff only and does not include representatives from the funding bodies, nor will it be influenced in any way by these funding bodies. Only the study researchers have access to the dataset.

### Confidentiality {27}

Information collected directly from participants will be in re-identifiable form and any information collected for, used in, or generated by this project will not be used for any other purpose. Only the study investigators will have access to study information.

### Plans for collection, laboratory evaluation, and storage of biological specimens for genetic or molecular analysis in this trial/future use {33}

Biological material will be collected in the form of biopsy material (muscle), plasma, and serum. The material will be stored in a project-specific research biobank at the Norwegian School of Sport Sciences. A small amount (≈100 mg) of muscle tissue will be obtained by the percutaneous needle technique under local anesthesia [21]. The muscle biopsy will be immediately snap frozen in liquid nitrogen and the samples will then be stored in – 80° until used for immunohistochemistry (5–10 μm sections will be cut and stained for relevant markers for capillaries per muscle fiber, satellite cell content, and macrophage infiltration), and protein quantification (samples will be determined for protein concentration by a protein assay, and immunoblotting will then be performed using relevant antibodies for markers of autophagy, mitophagy, and muscle catabolism) [22]. Blood samples (approximately 10 mL) will be collected and then analyzed with proteomics and metabolomics methods.

## Statistical methods

### Statistical methods for primary and secondary outcomes {20a}

Data will be analyzed using the IBM SPSS (Statistical Package for the Social Sciences) 26 statistical package for Windows (SPSS, Chicago, IL, USA). For the quantitative analysis, descriptive statistics, ANOVA, and chi-squared tests will be used to describe and compare baseline characteristics. For longitudinal analysis, linear mixed modeling adjusted for baseline values (T0) of the outcome will be used to detect any significant differences between the two groups from pre- to post-intervention as well as at follow-up 6 months following surgery. Group-by-time interaction will be included as a fixed effect. Corresponding effect sizes will also be calculated. Chi-squared tests and corresponding odds ratios will be calculated for hospitalization rates. All tests will be 2-tailed and statistical significance set at *p* ≤ 0.05.

### Interim analyses {21b}

An interim analysis will be performed after the first 10 participants have completed the exercise program and if it is registered that muscle mass is lower in the training group than in the control group the trial will be terminated.

### Methods for additional analyses (e.g., subgroup analyses) {20b}

For the primary outcome the model will, besides being adjusted for the baseline value, also be adjusted for diagnosis and type of surgery. Per protocol analysis for those who have adhered to the exercise program will be performed. Potential moderators of the exercise effect will be explored (e.g., age, sex, type of surgery, diagnosis). In addition, mediation analyses will be performed to explore potential underlying mechanisms of exercise effects (e.g., blood and muscle biomarkers). A subgroup analysis will also be performed to assess the rehabilitation effect only considering that some patients have a very limited time to exercise prior to surgery or may not be included until T2 due to very short time-to-surgery.

### Methods in analysis to handle protocol non-adherence and any statistical methods to handle missing data {20c}

Since the linear mixed model includes all available data (i.e., T1, T2, T3, and T4), intention-to-treat analysis is possible without imputing data.

### Plans to give access to the full protocol, participant-level data and statistical code {31c}

The full protocol and anonymized participant-level datasets used and analyzed during the current study are available from the corresponding author upon reasonable request.

## Oversight and monitoring

### Composition of the coordinating center and trial steering committee {5d}

The exercise testing is performed at the research groups’ own clinical gym: “Rörelseglädjelabbet” at Karolinska University Hospital, Stockholm, Sweden, and at the Norwegian site at the research lab facilities at the Norwegian School of Sports Sciences, Oslo, Norway. Both sites have all the necessary equipment to carry out advanced exercise tests including measurements of body composition and muscle ultrasound, and both labs have the necessary safety equipment (defibrillator, hygiene liquid dispensers, balance railings) and are in close proximity to medical staff if required. The multi-professional competence of the researchers means that the team is well-qualified to collect and analyze all data.

An established and working relationship to support this project exists with the Upper Gastrointestinal Unit at Cancer Theme at Karolinska University Hospital and at Oslo University Hospital which will play a key role in the inclusion of patients for this project. A working relationship also exists with The Swedish School for Sports and Health Sciences and The Norwegian School of Sports and Health Sciences, which has long experience with obtaining muscle biopsies from both healthy and clinical populations and to perform biochemical analyses. All biochemical methods planned to be performed in this study are set-up in these labs.

### Composition of the data monitoring committee, its role, and reporting structure {21a}

Since this randomized controlled exercise trial is not considered to be a large, complex, or high-risk clinical trial, such as those involving new drugs or medical devices, we deem that a DMC is not needed.

### Adverse event reporting and harms {22}

All AEs related to exercise or study measurements are recorded and reported to the sponsor. Participants in both groups are asked by the study personnel about exercise- and study measurement-related AEs in a standardized manner during all follow-up visits. In addition, the weekly telephone calls/video calls that are made to monitor exercise adherence for participants allocated to the exercise group will also be used to ask whether any potentially exercise-related AEs occurred during or since the last exercise session.

### Frequency and plans for auditing trial conduct {23}

The trial will be audited independently by investigators from the Clinical Trials Office at Karolinska University Hospital who will perform monitoring on-site or remotely according to good clinical practice (GCP) and national regulations.

### Plans for communicating important protocol amendments to relevant parties (e.g., trial participants, ethical committees) {25}

In the case changes in the protocol are necessary, relevant amendments will be made and submitted to the relevant ethics trial registration authorities and a resubmission with amendments to the study protocol will be submitted to Trials. All participants will provide informed written consent prior to their entry into the study.

### Dissemination plans {31a}

Results from the study will be published in scientific journals and popular science summaries will be sent to relevant patient organizations for dissemination. Results will be presented at international congresses and in the clinic and will also be communicated directly to and through the organizations that are responsible for developing and disseminating information on the National Physical Activity Guidelines and the Exercise Guidelines for the Prevention and treatment of Disease (FYSS textbook).

## Discussion

Developing countermeasures that deter muscle wasting in response to cancer could lead to major advances in improving the quality of life and extending survival in patients with upper GI and hepatobiliary cancers. Specifically, findings that will be generated from this study will lead to a better understanding of the skeletal muscle molecular characteristics in these fragile patient populations and shed light on the effects of BFR-T on skeletal muscle responses which is currently not understood. In fact, a major constraint in the development of therapies and countermeasures to prevent muscle wasting is the limited knowledge of the underlying mechanisms of cachexia derived from human studies, in large part due to the challenges of obtaining tissue samples from patients with cancer. The present study incorporates a novel approach in the cancer setting to counteract muscle atrophy through the incorporation of blood flow-restricted resistance training in the perioperative setting for patients with resectable pancreatic, biliary tract, and liver cancer.

Due to the COVID-19 pandemic, the study start was delayed for almost 1 year. The initial plan was to include a more homogenous group: patients diagnosed with pancreatic or biliary tract cancer undergoing a pancreaticoduodenectomy; however, there were major challenges with the inclusion rate resulting in the study taking longer and risking a loss of statistical power. Amendments were made to the original plan to expand the inclusion criteria to patients undergoing either pancreaticoduodenectomy or laparoscopic surgery and to also patients with malignancies in the liver amendable for surgical resection. These changes were approved by the Swedish Ethical Review Authority and the Norwegian Regional committees for medical and health research ethics. We are aware that this will result in a heterogeneous group of patients. There is considerably less risk of complications for patients undergoing laparoscopic surgery compared to a pancreaticoduodenectomy; however, these potential differences will be adjusted for in the statistical analysis. Moreover, time to surgery and post-surgery recovery are similar across the diagnoses. In this study, we decided to include any patient who is diagnosed with resectable pancreatic, biliary tract, or liver cancer regardless of time-to-surgery which means that some patients will only have time to perform (T0) before surgery, while some will perform both (T0) and (T1). In some cases, time-to-surgery is only a few days which means that inclusion is not possible at (T0) but will instead take place at (T2). The reason for including patients at (T2) is that time-to-surgery is significantly shorter at the Norwegian site; consequently, amendments to the original plan had to be made to accommodate for the reduced time-to-surgery. We will perform a subgroup analysis to evaluate the rehabilitation effect (T2 to T3). Other amendments to the original plan include the addition of digitally supervised exercise and an exercise diary to be able to monitor exercise adherence more efficiently.

We anticipate that adaptations to the exercise program will be required since these patient populations have a high disease burden and fluctuating health and performance status. Regular monitoring of the patients’ health status will be performed to adapt and personalize the exercise prescription. This means that the prescribed cuff pressure may decrease based on the self-reported rating of perceived exertion and the exercises may vary slightly based on post-surgery restrictions or complications. We believe that such adaptations to the initial planned training schedule are necessary for these specific populations.

To enhance adherence to the exercise regimen, the exercise sessions are personalized and home-based to avoid travel which has been shown to be a major barrier to exercise [23]. Additionally, utilizing a home-based training approach could offer a safer alternative for these patient populations, given their heightened susceptibility to infections. It is imperative for them to steer clear of infections to ensure timely eligibility for surgery.

In exercise-oncology trials, contamination is reported in 37% of cases. To mitigate this risk, we have implemented recommended measures, such as offering general exercise guidance along with an activity tracker to the control group [24]. While providing the activity tracker may potentially increase the physical activity levels of controls, research indicates that such proactive measures reduce the likelihood of drop-out and contamination [24].

In conclusion, in the PREV-Ex exercise trial, we are investigating the effects of blood flow-restricted resistance training in patients with pancreatic, biliary tract, and liver cancer in the perioperative setting on muscle thickness and muscle cross-sectional area, physical fitness, body composition, patient-reported and objective health-related outcomes, hospitalization, blood status, and skeletal muscle molecular characteristics. Findings from the study will shed light on the potential of a personalized exercise approach to act as a powerful strategy to counteract cancer-related muscle wasting. It will pave the way for the evidence-based practice that is needed to embed exercise into routine care for patients that are in crucial need for a strategy that can slow down the rate of skeletal muscle deterioration.

## Trial status

Protocol version number: 1.1 dated March 12, 2024.

Date when recruitment began: May 2022.

Date when recruitment is expected to be completed: November 1, 2024.

## Data Availability

The principal investigator archives the screening and inclusion log and other documents, such as informed consent, in a secure, fireproof cabinet marked as strictly confidential. All data will be stored at the local study center in an electronic logbook at Karolinska Institutet (KI ELN). Coded personal data can be stored in KI ELN since access to the data is limited to specifically authorized persons and the audit trail enables tracing of who viewed the results and when.

## Abbreviations

AE: Adverse event
ANOVA: Analysis of variance
BFR-T: Blood flow restriction training
UC: Usual care
CT: Computed tomography
DMC: Data monitoring committee
ECOG: Eastern Cooperative Oncology Group
EORTC: European Organization for Research and Treatment of Cancer
GCP: Good clinical practice
HRQoL: Health-related quality of life
PG-SGA: Patient-Generated Subjective Global Assessment scale
RCT: Randomized controlled trial
SAE: Serious adverse event
AOP: Arterial occlusion pressure
